# Correction: Rijpma et al. Wound and Short-Term Scar Outcomes of Meek Micrografting Versus Mesh Grafting: An Intra-Patient Randomized Controlled Trial. *Eur. Burn J.* 2025, *6*, 26

**DOI:** 10.3390/ebj6030039

**Published:** 2025-07-04

**Authors:** Danielle Rijpma, Karel Claes, Anouk Pijpe, Henk Hoeksema, Ignace De Decker, Jozef Verbelen, Matthea Stoop, Kimberly De Mey, Febe Hoste, Paul van Zuijlen, Stan Monstrey, Annebeth Meij-de Vries

**Affiliations:** 1 Alliance of Dutch Burn Care, Burn Center Beverwijk, Red Cross Hospital, Vondellaan 13, 1942 LE Beverwijk, The Netherlands; 2 Department of Plastic, Reconstructive and Hand Surgery, Vrije Universiteit Amsterdam, 1081 HV Amsterdam, The Netherlands; 3 Amsterdam Movement Sciences, Tissue Function and Regeneration, Boelelaan 1117, 1081 HV Amsterdam, The Netherlands; 4 Department of Plastic Surgery, Ghent University Hospital, C. Heymanslaan 10, 9000 Ghent, Belgium; 5 Burn Center, Ghent University Hospital, C. Heymanslaan 10, 9000 Ghent, Belgium; 6 Faculty of Medicine and Health Sciences, Ghent University Hospital, 9000 Ghent, Belgium; 7 Department of Plastic, Reconstructive and Hand Surgery, Red Cross Hospital, Vondellaan 13, 1942 LE Beverwijk, The Netherlands; 8 Department of Pediatric Surgery, Amsterdam UMC, Location AMC, Meibergdreef 9, 1105 AZ Amsterdam, The Netherlands; 9 Department of Surgery, Red Cross Hospital, Vondellaan 13, 1942 LE Beverwijk, The Netherlands

## Citation Correction

In the original publication [1], reference [21] ‘Schiefer, J.L.; Wergen, N.M.; Grieb, G.; Bagheri, M.; Seyhan, H.; Badra, M.; Kopp, M.; Fuchs, P.C.; Windolf, J.; Suschek, C.V. Experimental evidence for Parthanatos-like mode of cell death of heat-damaged human skin fibroblasts in a cell culture-based in vitro burn model. *Burns* **2024**, *50*, 1562–1577’, was incorrectly cited. The citation has now been replaced with the following:

Bagheri, M.; von Kohout, M.; Fuchs, P.C.; Seyhan, H.; Stromps, J.P.; Lefering, R.; Opländer, C.; Schiefer, J.L. How to evaluate scar colour after burn injuries—A clinical comparison of the Mexameter^®^ and the subjective scar assessment (POSAS/VSS). *Burns* **2024**, *50*, 691–701. doi:10.1016/j.burns.2023.11.010. PMID: 38097444.

The call-out of this reference in Discussion, Paragraph 2, has also been updated accordingly:

The differences in patient POSAS items at the 3-month follow-up, including ‘color’, ‘thickness’, ‘relief’, and ‘overall opinion’ between Meek micrografting and Mesh grafting, were all significantly in favor of Mesh grafting, ranging from 0.6 to 1.1 points. For the observer POSAS, the items ‘vascularity’, ‘thickness’, ‘relief’, ‘pliability’, ‘surface area’, and ‘overall opinion’ were significantly lower for Mesh grafting compared to Meek micrografting, ranging from 0.3 to 0.8 points. Legemate et al. (2024) demonstrated that the Minimal Clinical Important Difference (MCID) of the POSAS 2.0 was 0.39 points, meaning that the discovered differences of almost all POSAS items in this study are clinically meaningful [20]. In addition, the mean scar elasticity by the cutometer and mean melanin scores compared with normal skin by the dermaspectrometer showed a slight significant difference in favor of Mesh grafting. No significant differences were found for the patient POSAS items ‘pain’, ‘itch’, and ‘pliability’ and observer POSAS item ‘pigmentation’. Moreover, mean scarmaximal extension by the cutometer and erythema scores compared with normal skin by the dermaspectrometer showed no significant difference. Bagheri et al. (2024) [21] showed that the subjective and objective assessments of scar pigmentation could slightly differ. In this study, deviated scar pigmentation, compared to healthy skin, was more often observed with the subjective measurement tool (observer scale of the POSAS) compared to the objective measurement tool (mexameter). Therefore, the outcomes of objective (dermaspectrometer) and subjective (observer POSAS) measurement tools do not completely correlate with one other.

The authors state that the scientific conclusions are unaffected. This correction was approved by the Academic Editor. The original publication has also been updated.
